# Exploration of the profile-effect relationship of *Siraitia grosvenorii* aqueous extracts related to their laxative effect on the basis of gray correlation analysis

**DOI:** 10.1186/s12906-021-03388-x

**Published:** 2021-09-20

**Authors:** Wei Dong, Jia Zeng, Qin Wang, Xin Jiang, Ting Huang

**Affiliations:** 1 Department of Pharmacy, The Affiliated Hospital of Jiaxing University, Department of Pharmacy, The First Hospital of Jiaxing, Jiaxing, 314000 Zhejiang Province PR China; 2 NHC Key Laboratory of Reproduction Regulation, Shanghai Engineering Research Center of Reproductive Health Drug and Devices, Shanghai Institute of Planned Parenthood Research, Shanghai, 200032 PR China; 3 Department of Pharmacy, Guangxi University of Chinese Medicine, Nanning, 530001 Guangxi Province PR China

**Keywords:** *Siraitia grosvenorii*, Fingerprint, Laxative effect, Profile-effect relationship, Gray correlation analysis

## Abstract

**Background:**

*Siraitia grosvenorii* (binomial name *Siraitia grosvenorii* (Swingle) C. Jeffrey ex Lu et Z. Y. Zhang), also called Arhat Fruit or Monk’s Fruit, is a dried ripe fruit belonging to the Cucurbitaceae Family. *S. grosvenorii* has a long history of being used for constipation treatment in folk medicine. However, there are few studies where the laxative effect, related mechanisms, and active constituents of *S. grosvenorii* were investigated. This research explores the relationship between the common components and the laxative effect of aqueous extracts of S. *grosvenorii* from different habitats in China.

**Methods:**

The fingerprints of *S. grosvenorii* aqueous extracts from different habitats were established by HPLC. The constipation mice model was used to investigate the laxative effect of *S. grosvenorii* aqueous extracts. The motilin (MTL) level in mice serum, and the water content of the large and small intestines in mice were determined. The profile-effect relationship of *S. grosvenorii* aqueous extracts was preliminarily clarified using gray correlation analysis.

**Results:**

Nine common peaks were identified from the fingerprint of aqueous extracts of *S. grosvenorii*. The aqueous extracts obviously shortened the incubation period of defecation, and significantly increased the number of defecations, and the wet and dry weight of defecation in constipated mice. The profile-effect relationship indicated that seven common peaks were highly correlated with the effect of the incubation period of defecation, the number of defecations, and the wet and dry weight of defecation in mice.

**Conclusion:**

This work provides a promising method for the fingerprint establishment, pharmacodynamic evaluation, and quality control of *S. grosvenorii* on the basis of its profile-effect relationship.

**Supplementary Information:**

The online version contains supplementary material available at 10.1186/s12906-021-03388-x.

Constipation can lead to the continuous absorption of toxic substances, stimulate the local mucosa, and may cause malignant disease potentially in intestine. Natural products have long been and still are the source of treatments of different digestive disorders including cancer [1–5]. *S. grosvenorii* has a long history of being used for constipation treatment in folk medicine. In the present study, 624 ICR mice were approved to carry out the experiment by the Animal Research Ethics approval, and the animals were killed by carbon dioxide asphyxiation. This research explores the relationship between the common components and the laxative effect of aqueous extracts of S*. grosvenorii* from different habitats in China. *S. grosvenorii* from some places of origin were observed to significantly shorten the incubation period of defecation, and significantly increase the number of defecations, and the wet and dry weights of defecation in constipated mice. A potential explanation for the laxative effect of the aqueous extracts of *S. grosvenorii* was related to their ability to increase the MTL level in the serum of mice, and thus promote gastric emptying and intestinal propulsion. Further, it increased the water content of the small intestine, which supported its effect on bowel relaxation.

## Background

*Siraitia grosvenorii* (binomial name *Siraitia grosvenorii* (Swingle) C. Jeffrey ex Lu et Z. Y. Zhang), also called Arhat Fruit or Monk’s Fruit, is a dried ripe fruit belonging to the Cucurbitaceae Family [6]. As a famous edible and medicinal plant, *S. grosvenorii* has been cultivated for more than 200 years in China. It is abundant in many areas, such as northern Guangxi (mostly in the mountains of Guilin), and especially in Yongfu and Lingui counties (called “genuine medicinal materials”), as well as in Guangdong, Guizhou, Hunan, and Jiangxi [7]. Furthermore, it is a traditional export commodity of China and has been sold to more than 20 countries, such as America, Japan and several in south-east Asia [7]. In folk medicine applications, it shows definite curative effects in treating pertussis, chronic bronchitis, pharyngitis, and constipation.

Over the last 40 years, *S. grosvenorii* has been proven to contain many chemical constituents, including triterpene glycosides [8, 9], flavonoids [10, 11], proteins, vitamins, sugars, inorganic elements, and volatile oils [8]. Currently, cucurbitane triterpenoids, such as mogroside V, are considered to be the main active components of *S. grosvenorii*. Furthermore, modern pharmacological and clinical investigations have demonstrated that *S. grosvenorii* acts as an antitussive and expectorant [12–14], it stimulates the immune system [15, 16], offers liver protection [17, 18], eliminates free radicals [19], regulates blood sugar and blood fat levels [20–22], and has anti-inflammatory [23], anti-carcinogenic [24], and anti-fatigue effects [25]. In general, previous studies on *S. grosvenorii* mainly focused on the extraction and isolation of its chemical components and the screening and evaluation of its pharmacological activities. As previously described, *S. grosvenorii* has a long history of being used for constipation treatment in folk medicine. However, there are few studies where the laxative effect, related mechanisms, and active constituents of *S. grosvenorii* were investigated.

The present study is the first to clarify the profile-effect relationship of *S. grosvenorii* aqueous extracts related to their laxative effect. The relationship of the fingerprint of *S. grosvenorii* aqueous extracts with their laxative effect not only bridges the gap between the fingerprint and the observed pharmacodynamic effects, but also helps to establish the correlation between them. This study provides a promising method for the establishment of a fingerprint database, pharmacodynamic evaluation, and quality control of *S. grosvenorii* on the basis of its profile-effect relationship.

## Methods

### Chemicals and reagents

Mogroside V with a purity of 98% was purchased from Nanjing Spring & Autumn Biological Engineering Co., Ltd. (Nanjing, China). Chromatographic-grade acetonitrile and phosphoric acid were purchased from Sigma-Aldrich (St. Louis, Mo.). Diphenoxylate tablets were purchased from Changzhou Green Peak Environmental Protection Tech (50 mg/tablet, Changzhou, China). Mosapride citrate dispersible tablets were purchased from Chengdu Kanghong Pharmaceutical Group Co., Ltd. (5 mg/tablet, Chengdu, China). Activated charcoal was supplied by Chengdu Changzheng Glass Co., Ltd. (Chengdu, China). The mouse motilin (MTL) ELISA kit was supplied by Wuhan Boster Biological Technology Co., Ltd. (Wuhan, China). The other reagents were of analytical grade and commercially available.

### Animals

ICR mice with a body weight of 18–22 g were obtained from Hunan Slake Jingda Experimental Animal Co., Ltd. (Hunan, China). The animals were fed in a standard feeding room maintained from 18 to 26 °C at a relative humidity of 50–70%. The animals were killed by carbon dioxide asphyxiation after the experiments. All experiments were performed according to the guidelines for the care and use of animals established by Guangxi University of Chinese Medicine. All animal studies were in line with the guidelines of the Animal Ethics Committee of Guangxi University of Chinese Medicine.

### Collection and identification of *S. grosvenorii*

**Fig. 1:**
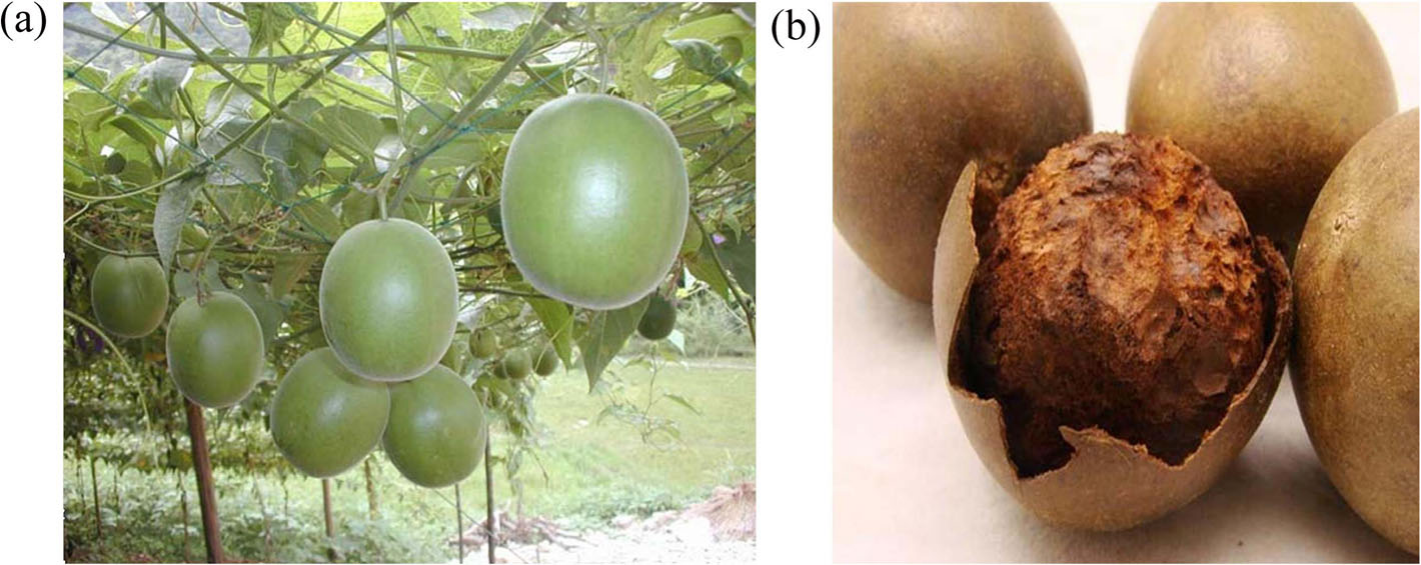
Appearance of *S. Grosvenorii* (**a**) fresh fruits; (**b**) dried fruits

A total of 25 batches of *S. grosvenorii* from different ecological regions or from the same region with different growth phases, breeding type, and sizes were collected in Guangxi and Hunan Provinces, China. The samples were identified as the fruit of *Momordica grosvenorii* Swingle by Professor Tian Hui from Guangxi University of Chinese Medicine. The appearance of *S. grosvenorii* is shown in Fig. 1 and the detailed sample information is summarized in Table 1 (Supplementray file).

### Preparation of *S. grosvenorii* aqueous extracts

Water extraction and an alcohol precipitation method were used to prepare *S. grosvenorii* aqueous extracts as follows. First, the herbs were weighed and broken into small pieces. Then, they were decocted three times with 10, 8, and 5 fold water for 1.5 h, 1 h, and 1 h, respectively. Before the first extraction, the herbs were soaked for 0.5 h in water. After decocting, the extracted solutions were blended, filtered, and concentrated to a relative density of 1.05 g/mL. Then an equal volume of 95% ethanol was added and the mixture was refrigerated for 48 h undisturbed to allow the solid components to deposit at the bottom of the container. Finally, the ethanol-deposition components were filtered and concentrated to a paste for analysis.

### HPLC method for fingerprinting the *S. grosvenorii* aqueous extracts

The fingerprint of the *S. grosvenorii* aqueous extracts was established using an Agilent 1100 series HPLC system (Agilent Technologies, Santa Clara, CA, USA). Mogroside V and S. *grosvenorii* aqueous extracts were dissolved in methanol to prepare the reference solution and the test solution, respectively. A ZORBAX SB-C18 column (4.6 × 150 mm, 5 μm) maintained at a flow rate of 0.8 mL/min and 20 °C was used for the separation. The mobile phase consisted of 0.05% phosphoric acid (A) and acetonitrile (B) (0–8 min, 3.0–13.5% B; 8.0–35.0 min, 13.5–35.0% B; 35.0–45.0 min, 35.0% B). A 10 μL aliquot of the sample was injected into the system and the components detected at 203 nm. The total run time was 45 min.

### Investigation of the laxative effect of *S. grosvenorii* aqueous extracts

A mouse constipation model induced by diphenoxylate was used to investigate the laxative effect of *S. grosvenorii* aqueous extracts from different areas of Guangxi and Hunan Provinces, China. Male ICR mice were randomly divided into 28 groups, 18 mice in each group. The model groups were orally administered a diphenoxylate solution at a dose of 10 mg/kg and the negative control group was orally administered an equal volume of pure water. Before the experiment, the animals were fasted for 12 h with access to drinking water. Except for the negative control group, the other mice were given 10 mg/kg diphenoxylate solution by gavage (0.2 mL/10 g, volume/body weight) once a day for 3 consecutive days to construct the constipation model. A total of 40 min after the diphenoxylate dose, the model mice were treated with an equal volume of pure water, 10 mg/kg mosapride solution, and 30 g/kg *S. grosvenorii* aqueous extracts from different areas. After another 40 min, the mice were orally administered a 5% activated charcoal solution. The incubation period of defecation, the number of defecations, the wet weight of defecation, and the dry weight of defecation over the following 3 h were then recorded. The animals were killed by carbon dioxide asphyxiation when the experiments were finished.

### Determination of the MTL level in the serum of normal mice

A total of 80 male mice weighing 18–22 g were randomly divided into 5 groups with 16 per group. The groups included the blank control group, the positive control group, and the *S. grosvenorii* aqueous extract group treated with low, middle, and high doses (15 g/kg, 30 g/kg, and 45 g/kg, respectively, administered once a day for 10 consecutive days). At 1 h after the last administration, 1 mL of blood was collected from the eyeball and placed in a centrifugation tube. The animals were killed by carbon dioxide asphyxiation. The blood coagulated naturally at room temperature for 10–20 min, and then was centrifuged (3000 rpm) at 4 °C for 20 min. The supernatant was then separated and the mouse serum was prepared.

A MTL ELISA kit was used to determine the MTL level in the mouse blood serum. The level of MTL was determined by a double-antibody sandwich method. The purified mouse MTL antibody was coated on a microporous plate to create a solid-phase antibody. MTL was added to the coated plate, and a horseradish peroxidase (HRP)-labeled MTL antibody was added to form the antibody, antigen, and enzyme-labeled antibody complex. After thorough washing, the substrate tetramethylbenzidine (TMB) was added to determine the amount of MTL antigen captured. TMB was first converted to blue by HRP, and then to yellow in the presence of acid. There was a positive correlation between the color and MTL level in the samples. The optical density (OD) value was measured at 450 nm using a microplate reader within 15 min after adding the termination solution. The MTL level in the serum of mice was calculated by a standard curve. The operation steps were in strict accordance with the instructions of the MTL ELISA Kit.

### Determination of the water content in the small and large intestines of normal mice

The effect of *S. grosvenorii* aqueous extracts on the water content of the large and small intestines in normal mice were tested by a weighing method. A total of 40 mice, half male and half female, were randomly divided into the blank control group and the *S. grosvenorii* aqueous extracts group with 20 in each group. After 18 h of fasting, one group was treated with *S. grosvenorii* aqueous extracts at a dose of 45 g/kg and the blank control group was administered an equal volume of distilled water. After 75 min, the mice were put to death by carbon dioxide asphyxiation. The abdominal cavity was opened and the pylorus, ileocecal, and rectum were ligated. Next, the ligated pylorus, ileocecal and rectum were cut to ensure that the water in the intestinal cavity did not flow out. The wet weights of the large and small intestines of each mouse were determined and the intestines were stored on a square tray. The dry weight was weighed after heating at 80 °C for 6 h. The water content in the small and large intestines of mice was calculated according to Formula (1).

1 $$ \mathrm{Water}\ \mathrm{content}\ \left(\mathrm{g}\right)=\mathrm{Wet}\ \mathrm{weight}\ \left(\mathrm{g}\right)-\mathrm{Dry}\ \mathrm{weight}\ \left(\mathrm{g}\right) $$

### Profile-effect relationship investigation

The profile-effect relationship of *S. grosvenorii* aqueous extracts related to their laxative effect was investigated using a gray correlation analysis. The detailed steps were as follows.

#### Step 1: selection of the reference and comparison sequences

According to gray system theory, the chemical compositions of *S. grosvenorii* aqueous extracts were regarded as a whole. Different common peaks were regarded as the subsystems, and the relative peak area of each characteristic component was calculated as a gray element. The laxative effect of *S. grosvenorii* aqueous extracts including the incubation period of defecation, the number of defecations, the wet weight of defecation, and the dry weight of defecation were treated as the reference sequence denoted by X0 as shown in Formula (2). The main characteristic components of *S. grosvenorii* aqueous extracts were treated as the comparison sequence denoted by Xi as shown in Formula (3).

2 $$ \mathrm{X}0=\left[\mathrm{X}0\ \left(\mathrm{l}\right),\mathrm{X}0\ (2),\mathrm{X}0\ (3),\dots \mathrm{X}0\ \left(\mathrm{k}\right)\right] $$

Where k represents *Siraitia grosvenorii* from different regions, k = 25.

3 $$ \mathrm{Xi}\ \left(\mathrm{k}\right)=\left[\mathrm{Xi}\ (1),\mathrm{Xi}\ (2),\mathrm{Xi}\ (3),\dots \mathrm{Xi}\ (25)\right] $$

#### Step 2: calculation of the gray correlation coefficient

According to the determined reference and comparison sequences, the calculation formula of the gray correlation coefficient is shown in Formula (4).

4 $$ {\varepsilon}_i(k)=\frac{\begin{array}{c}\mathit{\min}\\ {}i\end{array}\begin{array}{c}\ \mathit{\min}\\ {}k\end{array}\left|X0(k)- Xi(k)\right|+\uprho \cdot \begin{array}{c}\mathit{\max}\\ {}i\end{array}\begin{array}{c}\ \mathit{\max}\\ {}k\end{array}\left|X0(k)- Xi(k)\right|}{\left|X0(k)- Xi(k)\right|+\uprho \cdot \begin{array}{c}\mathit{\max}\\ {}i\end{array}\begin{array}{c}\ \mathit{\max}\\ {}k\end{array}\left|X0(k)- Xi(k)\right|} $$

Where k represents *S. grosvenorii* from different regions, X_i_ represents the main characteristic components of *S. grosvenorii* aqueous extracts, *ε*_*i*_ represents the correlation coefficient between the comparison and the reference sequences of the k-th batch of *S. grosvenorii*. $$ {\displaystyle \begin{array}{c}\mathit{\min}\\ {}i\end{array}}{\displaystyle \begin{array}{c}\ \mathit{\min}\\ {}k\end{array}}\left|X0(k)- Xi(k)\right| $$ represents the minimum difference between the comparison and the reference sequences, denoted by △min.

$$ {\displaystyle \begin{array}{c}\mathit{\max}\\ {}i\end{array}}{\displaystyle \begin{array}{c}\ \mathit{\max}\\ {}k\end{array}}\left|X0(k)- Xi(k)\right| $$ represents the maximum difference between the comparison and the reference sequences, denoted by △max. |*X*0(*k*) − *Xi*(*k*)| represents the absolute difference between the comparison and the reference sequences, denoted by △i (k). ρ is the resolution coefficient, which reduces the distortion in calculation caused by $$ {\displaystyle \begin{array}{c}\mathit{\max}\\ {}i\end{array}}{\displaystyle \begin{array}{c}\ \mathit{\max}\\ {}k\end{array}}\left|X0(k)- Xi(k)\right| $$ and thus improves the significance of the difference between the correlation coefficients. The value range of ρ is usually between 0 and 1. In this study, it was set as 0.5.

#### Step 3: Calculation of the gray correlation grade

The gray correlation grade is the arithmetic mean of the gray correlation coefficient shown in Formula (5).

5 $$ \mathrm{ri}=\frac{1}{N}\sum \limits_{k=1}^n\varepsilon i(k) $$

The relevancy grade of the reference sequence and each comparison sequence were rearranged into a row according to the relevancy order, which reflects the correlation grade of each comparison sequence to the reference sequence. The higher the correlation grade is, the closer the relationship is between the two characteristic components of *S. grosvenorii* aqueous extracts. As previously described, the laxative effect of *S. grosvenorii* aqueous extracts were treated as the reference sequence, and the nine common peaks (P1, P2, P3, P4, P5, P6, P7, P8, and P9) were treated as the comparison sequence. They were analyzed by the gray correlation method.

#### Step 4: dimensionless treatment

Since each sequence has different units or dimensions, each sequence should be dimensionless before performing the correlation analysis so as to achieve dimensional consistency. In the present study, a mean transformation was used to form new data columns. Namely, the average value of each sequence data was calculated, and then it was divided by the raw data in the corresponding sequence.

#### Step 5: calculation of the absolute difference

The absolute difference △i (k) between the reference sequence and the comparison sequence was calculated according to the dimensionless value as shown in Formula (6).

6 $$ \triangle \mathrm{i}\left(\mathrm{k}\right)=\left|X0(k)- Xi(k)\right|. $$

#### Step 6: calculation of the correlation coefficients between the characteristic peaks and the laxative effect

According to the calculation results from step 5, the value of △max and △min (△max = 8.515745, △min = 0) were substituted into Formula (4). Then the correlation coefficients between the characteristic peaks and the laxative effect were obtained.

### Data analysis

In the gray correlation analysis, data were processed and analyzed using the data analysis function of Microsoft Excel.

## Results

### HPLC fingerprint of *S. grosvenorii* aqueous extracts from different batches

**Fig. 2:**
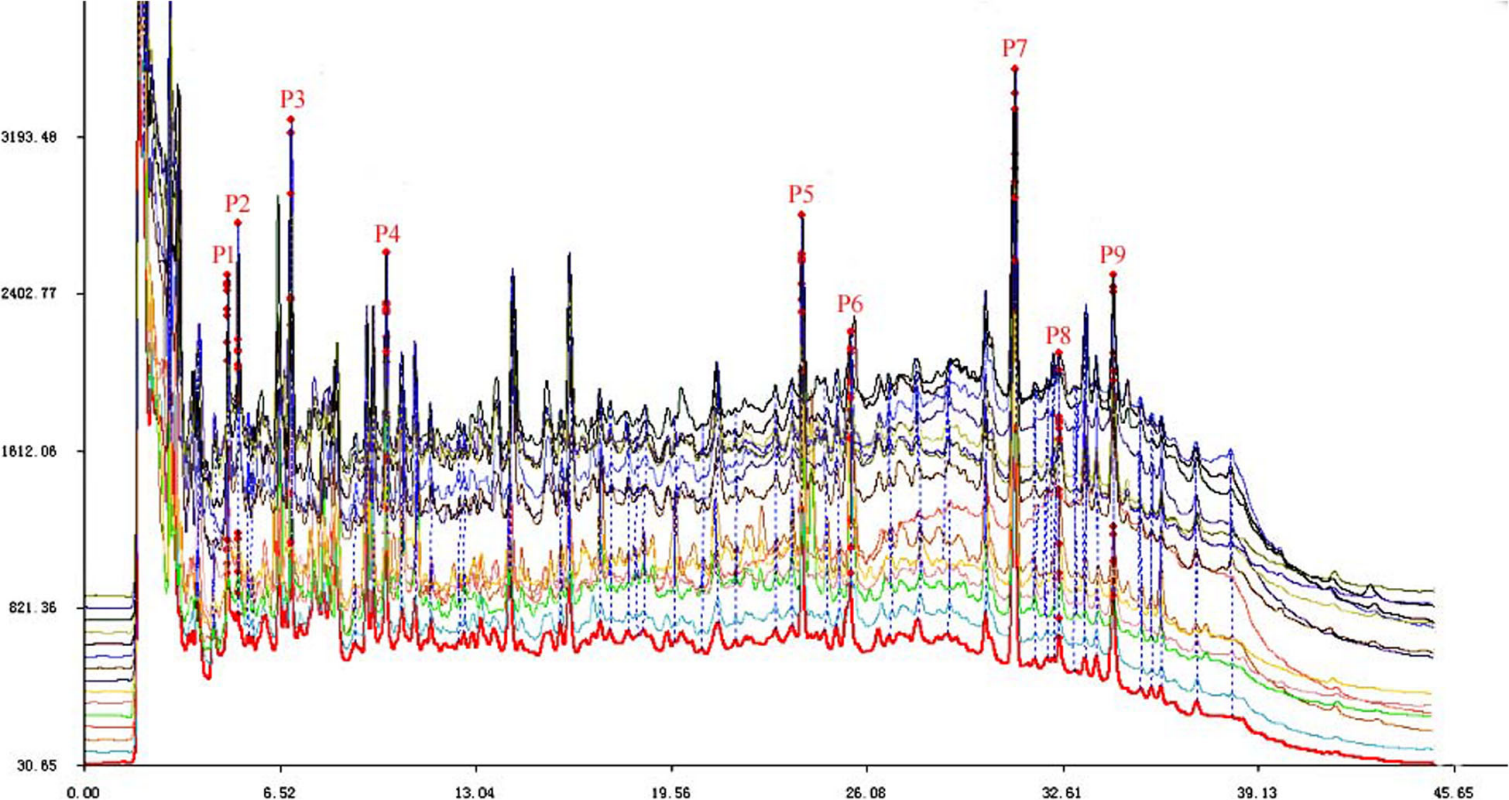
HPLC Fingerprint of *S. grosvenorii* aqueous extracts from different batches

Using the established chromatographic conditions, the HPLC fingerprint of 25 batches of *S. grosvenorii* aqueous extract and the common peaks are shown in Fig. 2. In the *S. grosvenorii* aqueous extracts from different regions, nine common fingerprint peaks were identified and named Peak 1 (P1), Peak 2 (P2), Peak 3 (P3), Peak 4 (P4), Peak 5 (P5), Peak 6 (P6), Peak 7 (P7), Peak 8 (P8), and Peak 9 (P9) according to the order of their retention times. Among them, P7 was determined to be mogroside V based on a comparison of the retention time and ultraviolet spectrum of a reference sample of mogroside V. The identities of the other eight common peaks remain unknown.

### The laxative effect of *S. grosvenorii* aqueous extracts from different batches

**Fig. 3:**
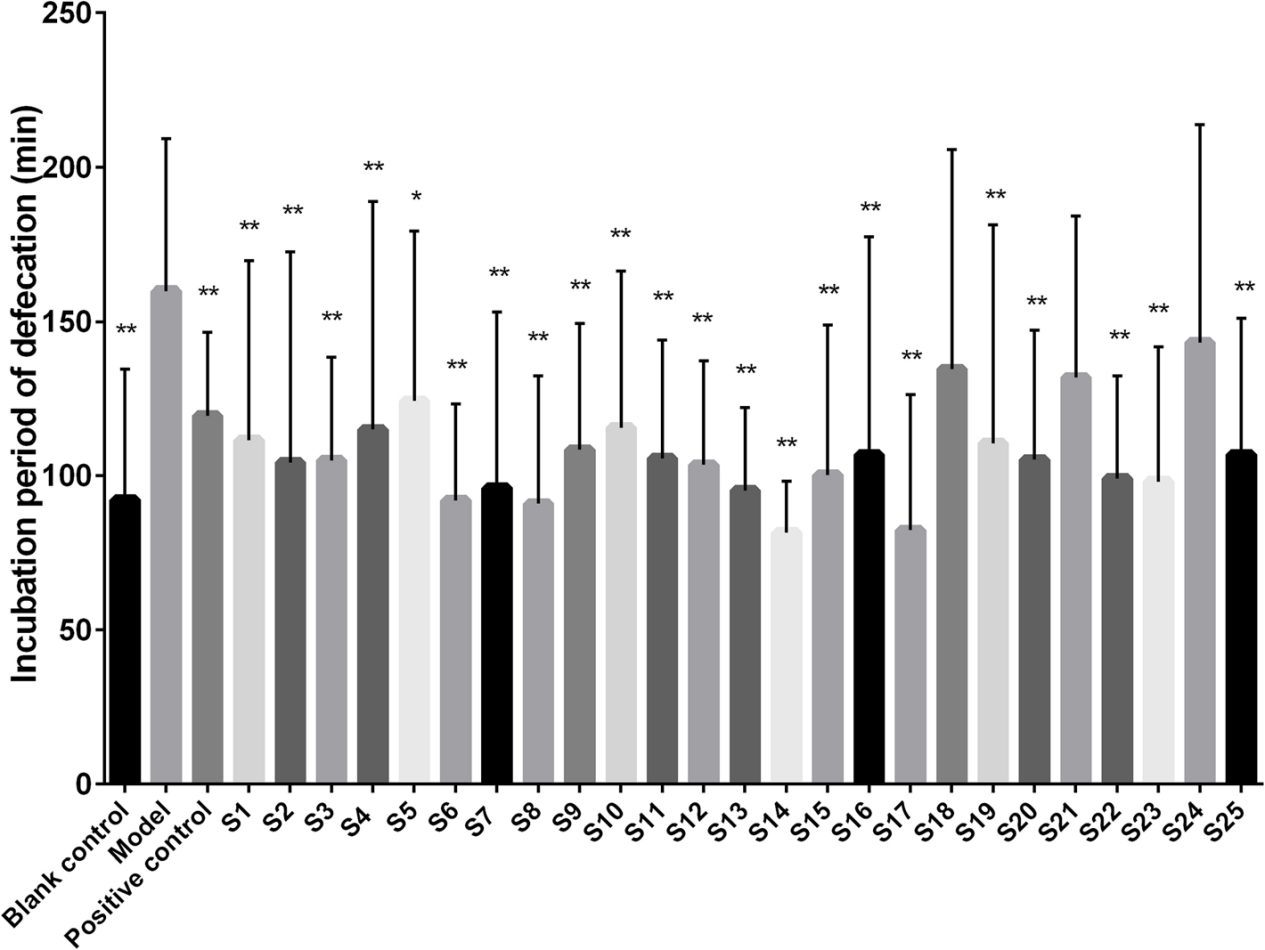
Effect of *S. grosvenorii* aqueous extracts on the incubation period of defecation in mice with constipation (*P < 0.05, **P < 0.01)

**Fig. 4:**
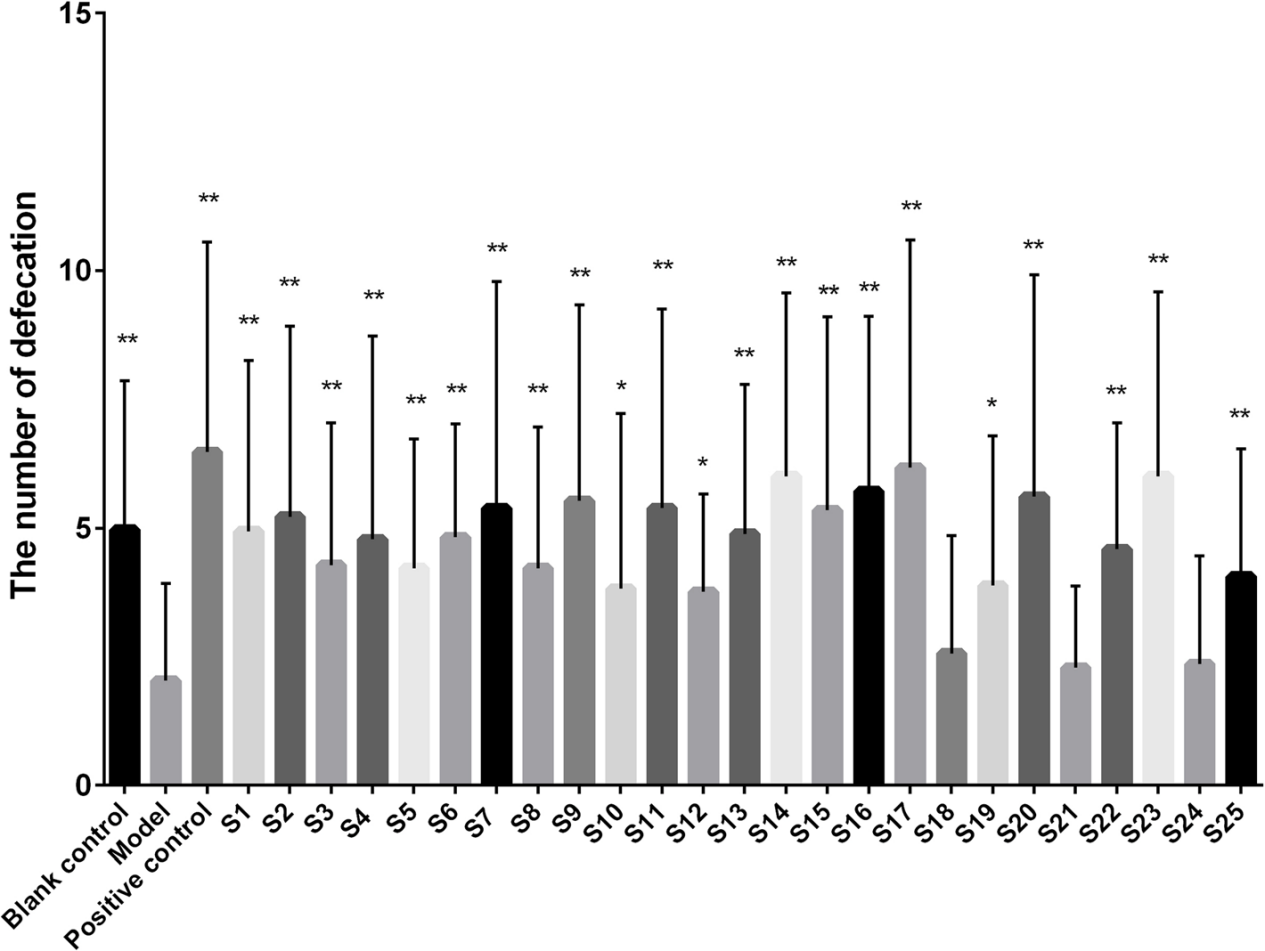
Effect of *S. grosvenorii* aqueous extracts on the number of defecation in mice with constipation (*P < 0.05, **P < 0.01)

**Fig. 5:**
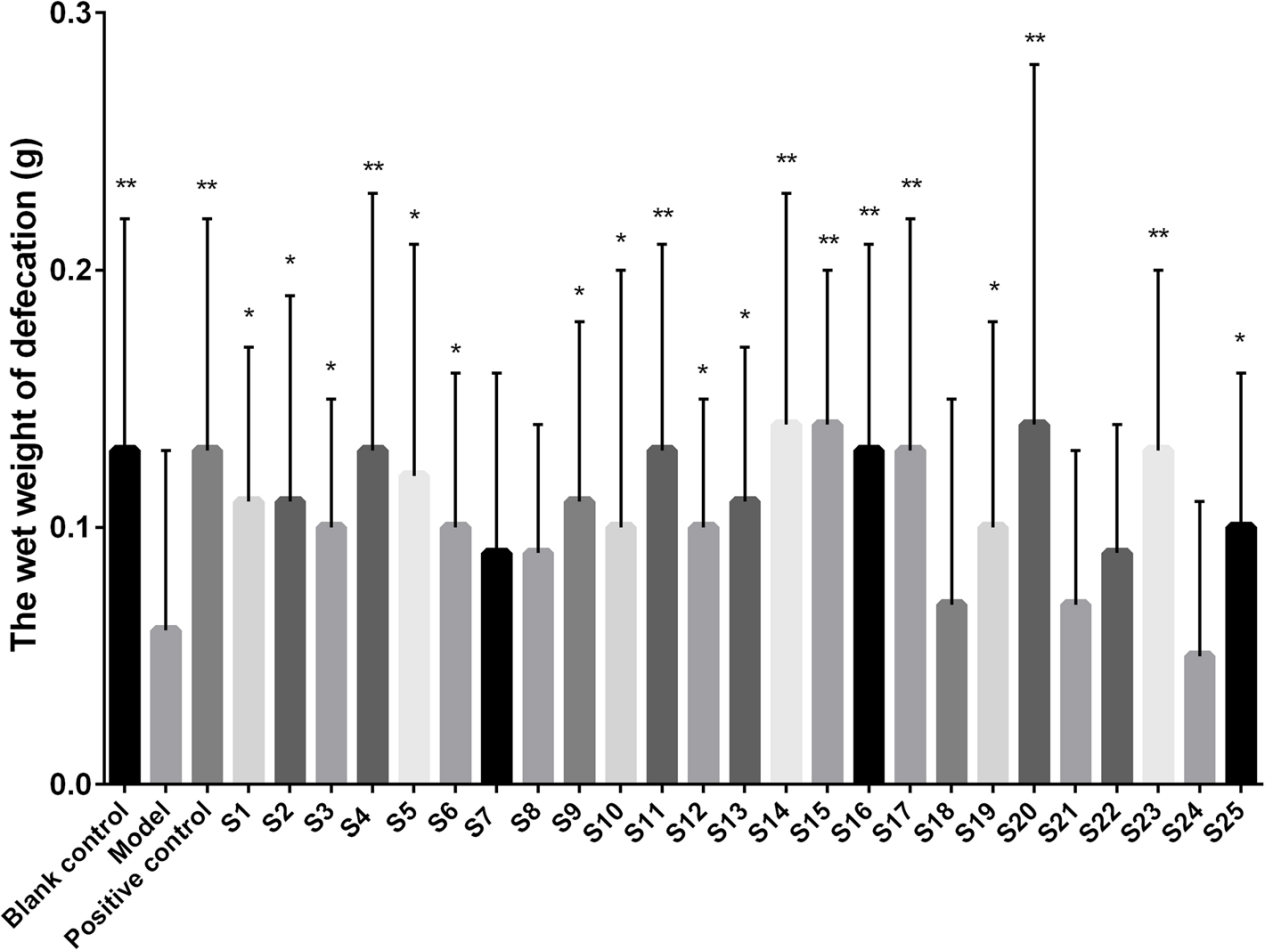
Effect of *S. grosvenorii* aqueous extracts on the wet weight of defecation in mice with constipation (*P < 0.05, **P < 0.01)

**Fig. 6:**
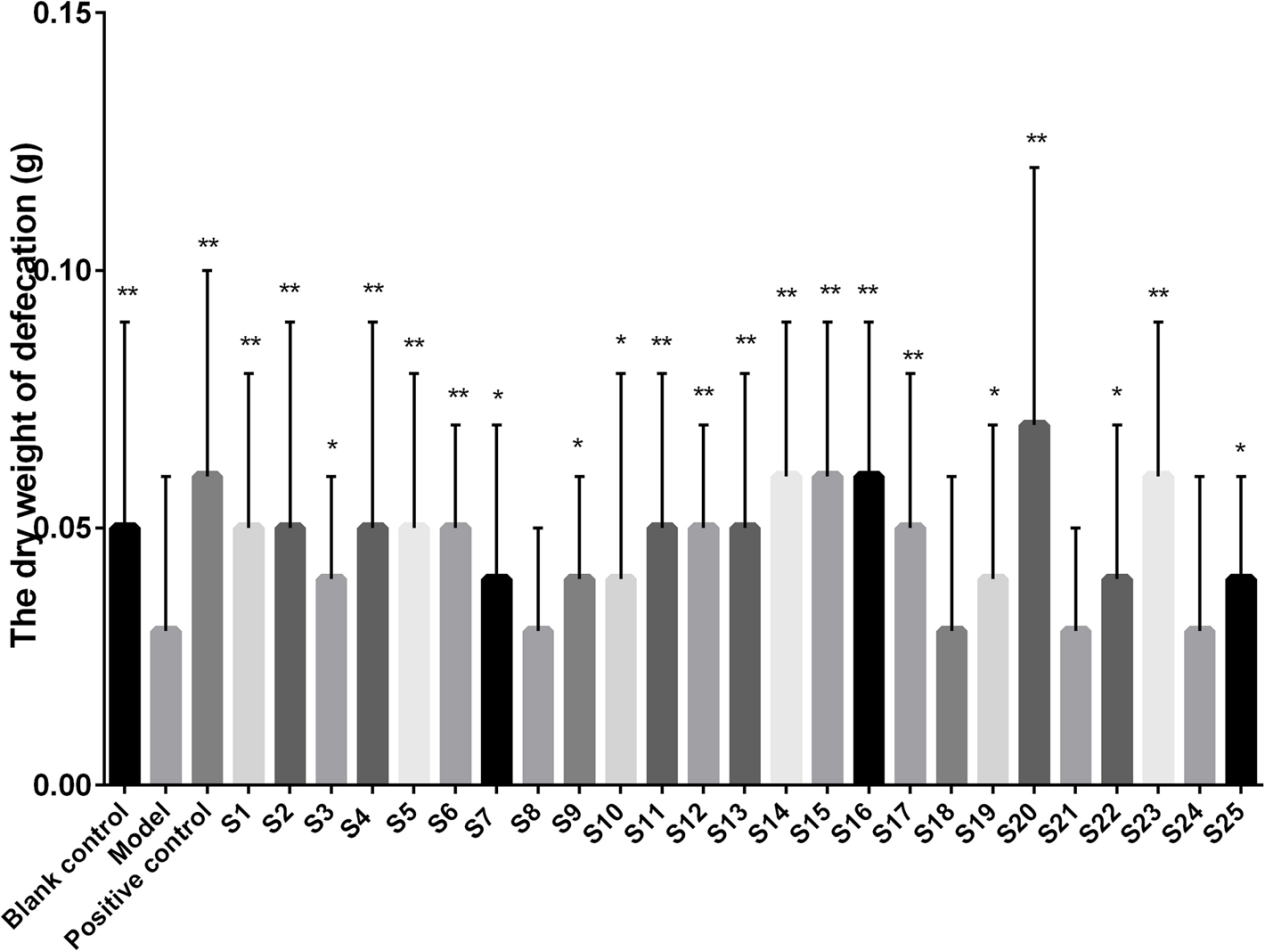
Effect of *S. grosvenorii* aqueous extracts on the dry weight of defecation in mice with constipation (*P < 0.05, **P < 0.01)

The effects of *S. grosvenorii* aqueous extracts from different batches on the constipation model of mice induced by diphenoxylate are shown in Figs. 3, 4, 5 and 6. Compared with the blank control group, the incubation period of defecation in the model group was significantly prolonged (*P* < 0.01), while the number of defecations, the wet weight of defecation, and the dry weight of defecation were significantly reduced (*P* < 0.01), indicating that the constipation model was successfully constructed. *S. grosvenorii* aqueous extracts from different batches exhibited different therapeutic effects on mice with constipation. Figure 3 shows that S1–S4, S6–S17, S19–S20, S22–S23, and S25 obviously shortened the incubation period of defecation in constipated mice (*P* < 0.01), and were equivalent or more effective than the positive control. Figure 4 shows that S1–S9, S11, S13–S17, S20, S22–S23, and S25 significantly increased the number of defecations in constipated mice (*P* < 0.01). Figure 5 shows that S4, S11, S14–S17, S20, and S23 significantly increased the wet weight of defecation in constipated mice (*P* < 0.01), of which S4, S11, S14–17, and S20 were equivalent or more effective than the positive control. Figure 6 shows that S1–S2, S4–S6, S11–S17, S20, and S23 significantly increased the dry weight of defecation in constipated mice (*P* < 0.01), of which S14–S16, S20, and S23 were equivalent or more effective than the positive control. In general, S4, S11, S14–S17, S20, and S23 all obviously shortened the incubation period of defecation (P < 0.01), and significantly increased the number of defecations, the wet weight of defecation, and the dry weight of defecation in constipated mice (P < 0.01). The large differences in the laxative effect of *S. grosvenorii* aqueous extracts from different batches may be attributed to the different metabolic fingerprints, and the latter were affected by different ecological environments or different growth phases, breeding type and/or sizes.

### Effect of *S. grosvenorii* aqueous extracts on the MTL level in serum of normal mice

**Fig. 7:**
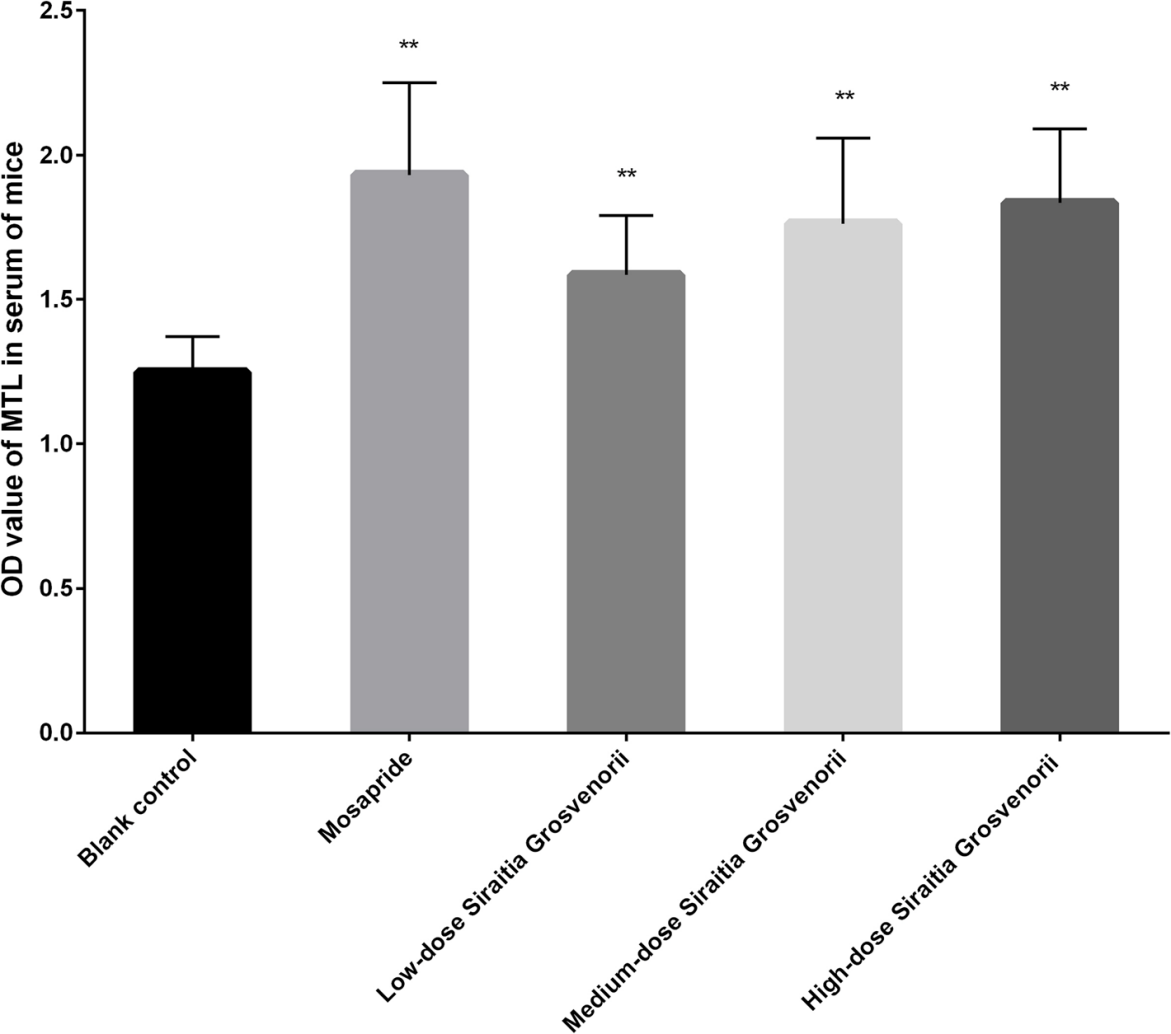
Level of MTL in serum of mice treated with different dose of *S. grosvenorii* aqueous extracts (**P < 0.01)

The effect of *S. grosvenorii* aqueous extracts on the MTL level in the serum of normal mice is shown in Fig. 7. Mosapride at a dose of 10 mg/kg and *S. grosvenorii* aqueous extracts, whether administered at a low, moderate or high dose, significantly increased the content of MTL in the serum of normal mice (*P* < 0.01). The effect of *S. grosvenorii* aqueous extracts on the MTL level in the serum of normal mice was dose-dependent and the effect of the high dose group was similar to that of mosapride. The present results indicated that *S. grosvenorii* aqueous extracts promoted gastric emptying and intestinal peristalsis.

### Effect of *S. grosvenorii* aqueous extracts on water absorption of large and small intestines in normal mice

**Fig. 8:**
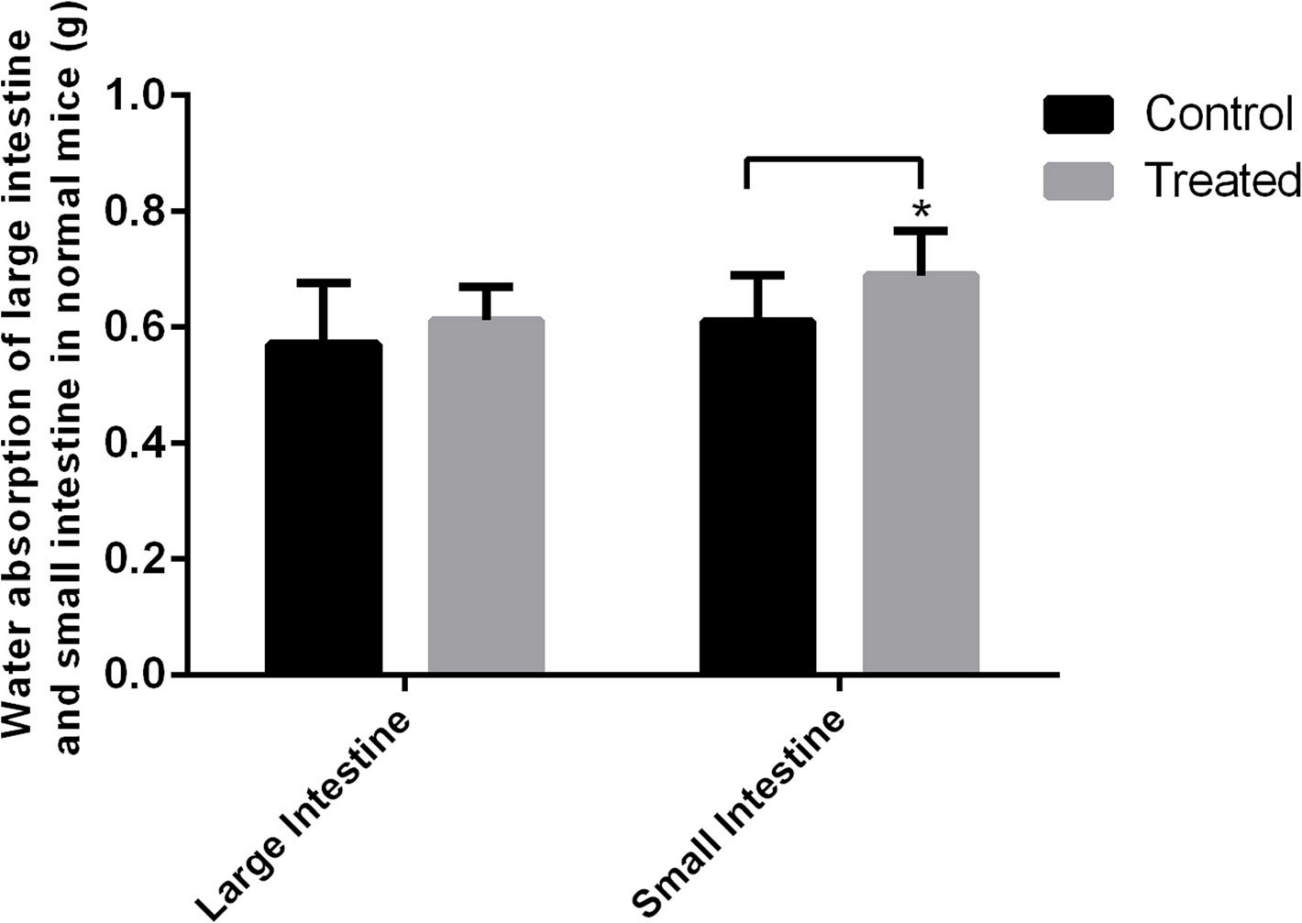
Effect of *S. grosvenorii* aqueous extracts on water absorption of large and small intestines in normal mice (*P < 0.05)

The effect of *S. grosvenorii* aqueous extracts on the water absorption of the large and small intestines in normal mice is shown in Fig. 8. Compared with the blank control group, the high dose of *S. grosvenorii* aqueous extracts increased the water content of the large intestine in normal mice, but the difference was not significant (*P* > 0.05). By comparison, the same dose of *S. grosvenorii* aqueous extracts increased the water content of the small intestine in normal mice with a significant difference (*P* < 0.05). These results suggested that one of the primary mechanisms of *S. grosvenorii* aqueous extracts related to their laxative effect was to increase the water content of the small intestine.

### Profile-effect relationship investigation

**Fig. 9:**
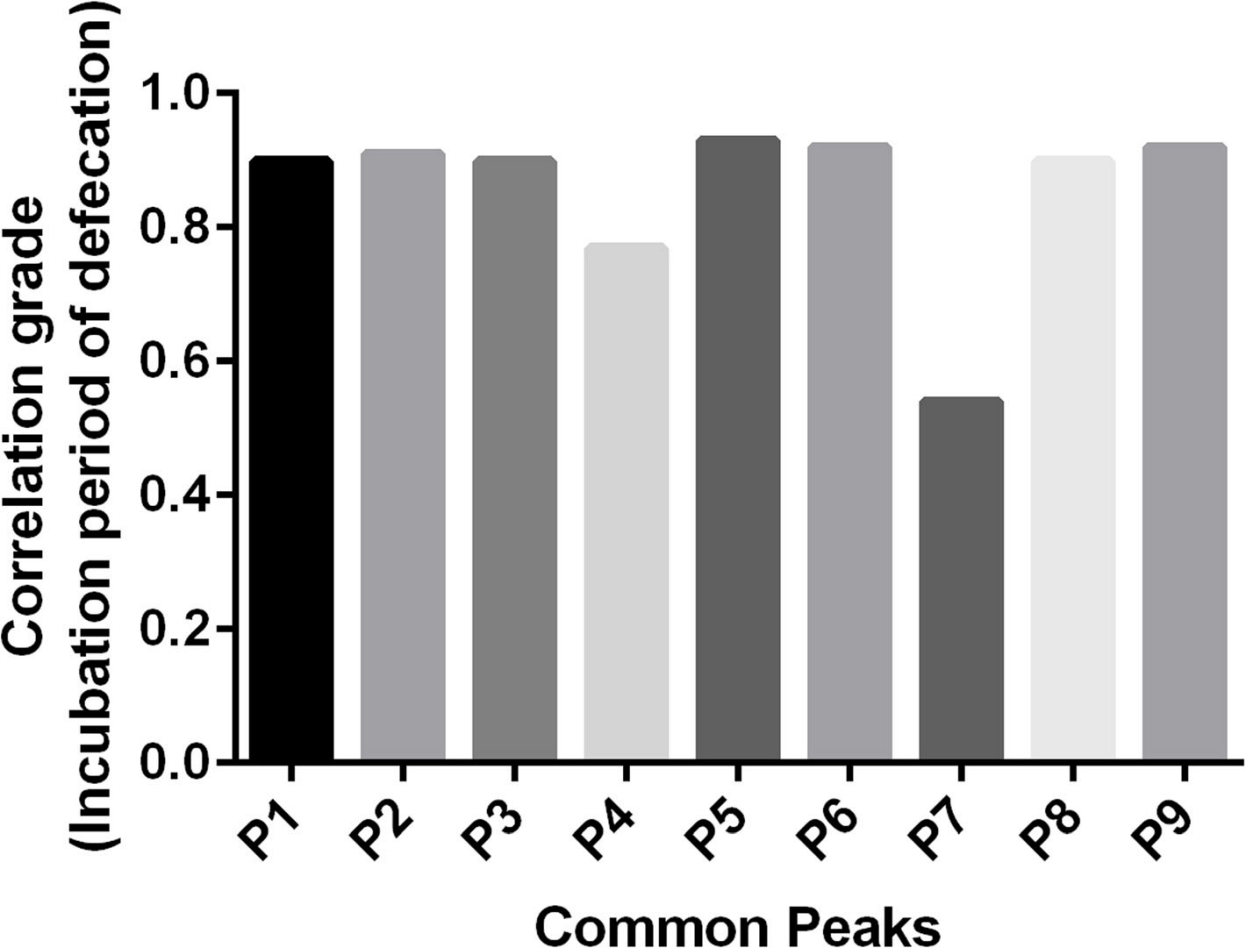
Correlation grade between the incubation period of defecation in mice with constipation and the common peaks in *S. grosvenorii* aqueous extracts

**Fig. 10:**
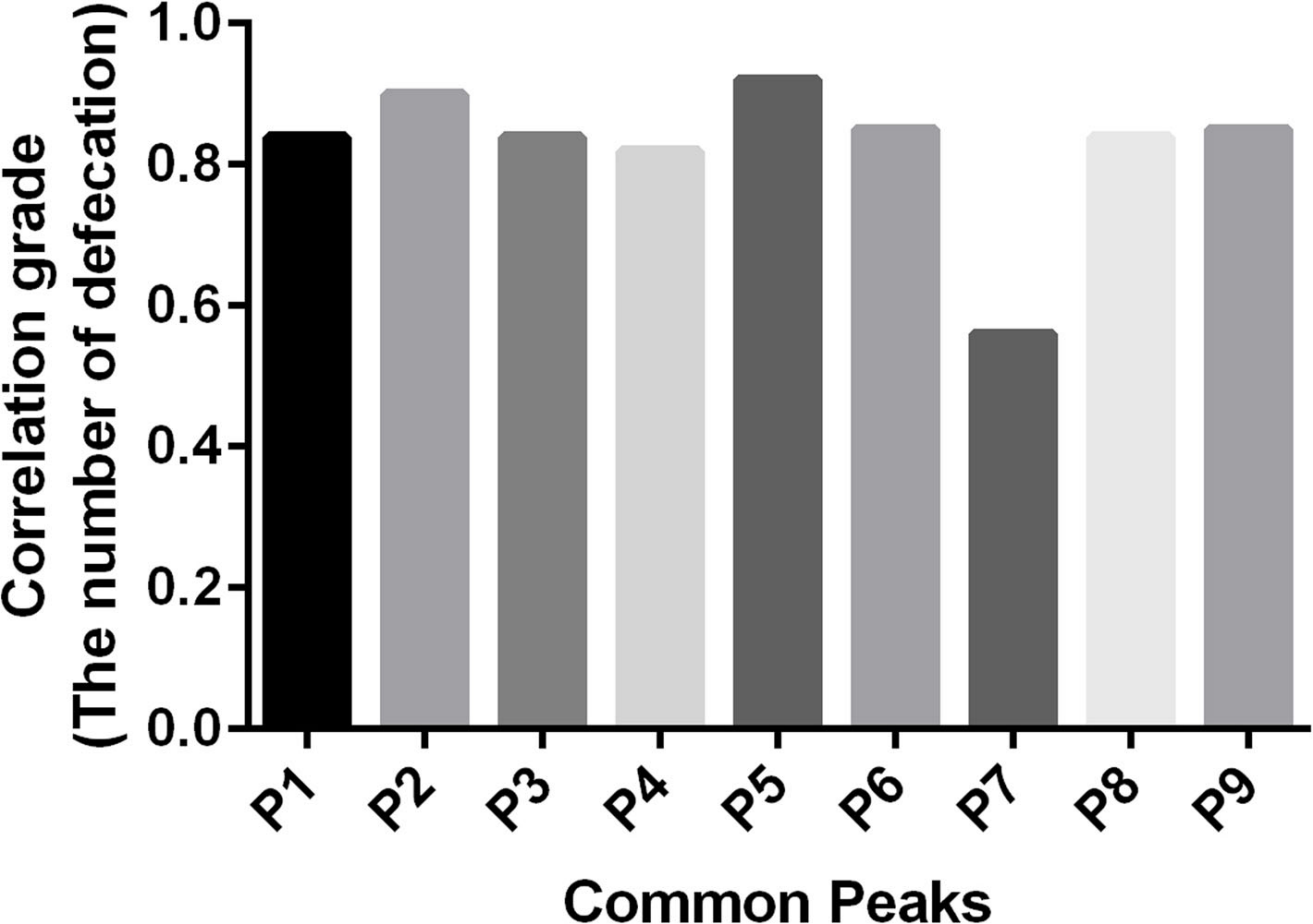
Correlation grade between the number of defecation in mice with constipation and the common peaks in *S. grosvenorii* aqueous extracts

**Fig. 11:**
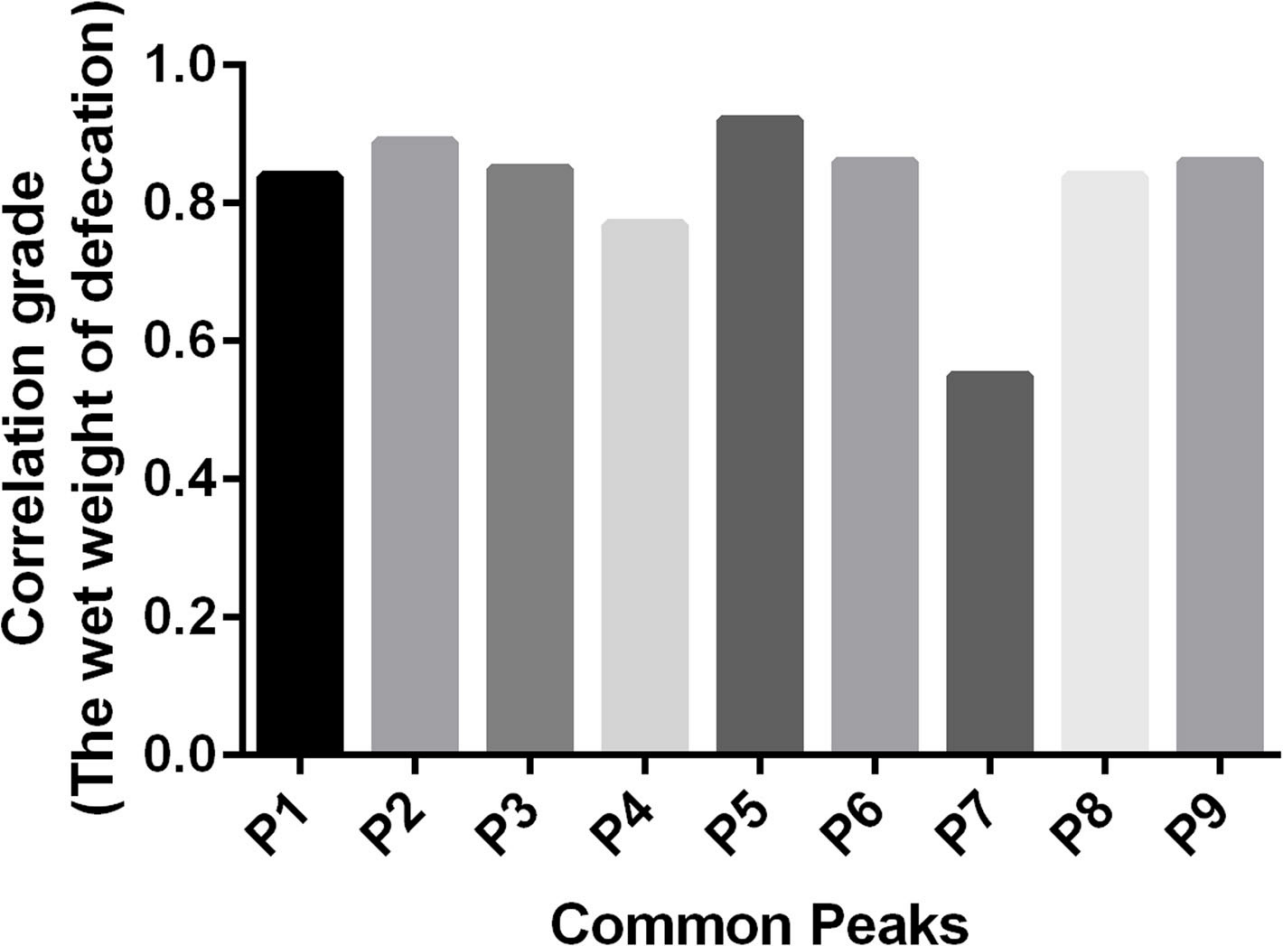
Correlation grade between the wet weight of defecation in mice with constipation and the common peaks in *S. grosvenorii* aqueous extracts

**Fig. 12:**
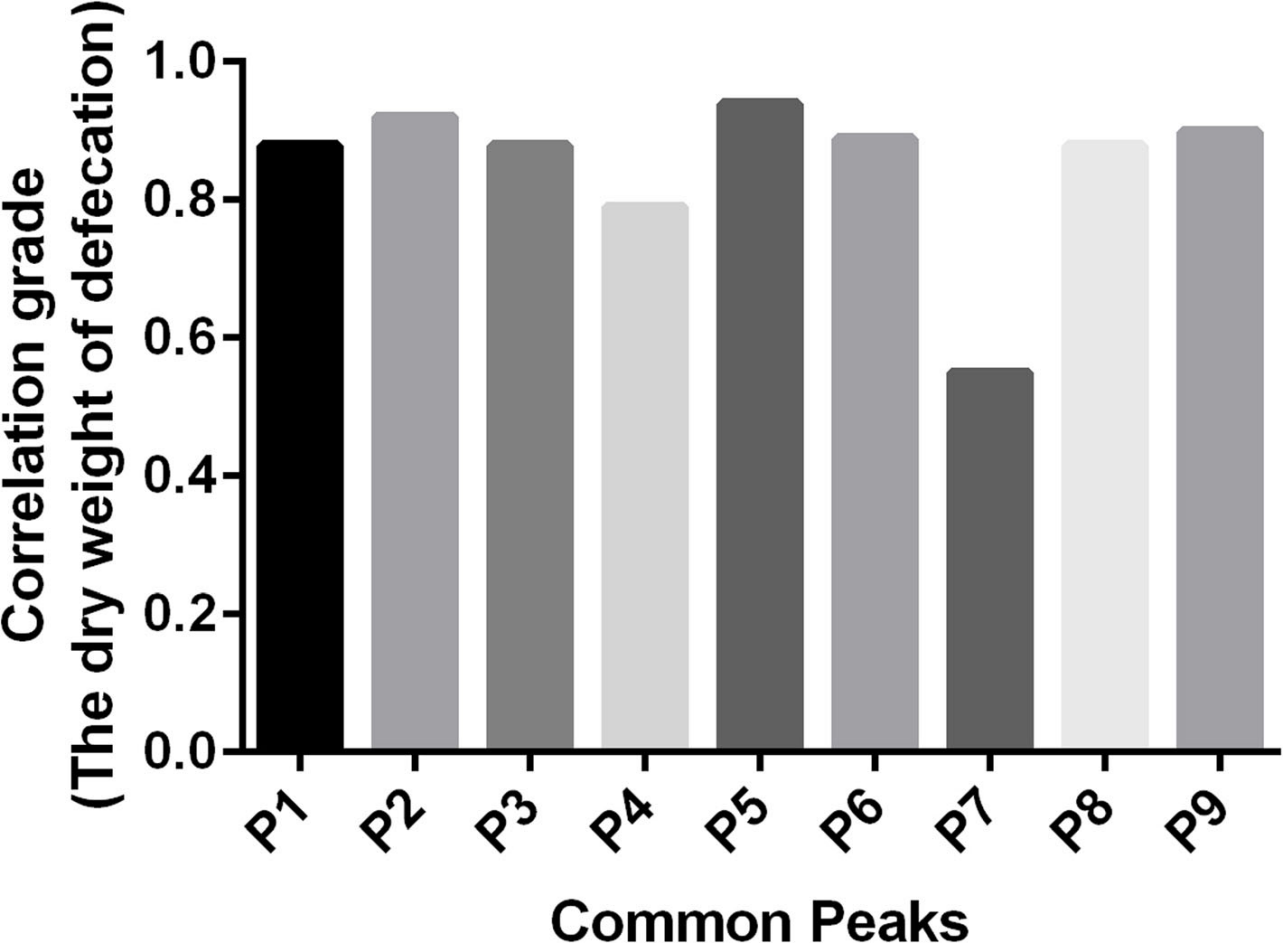
Correlation grade between the dry weight of defecation in mice with constipation and the common peaks in *S. grosvenorii* aqueous extracts

The profile-effect relationship between the nine common peaks of *S. grosvenorii* aqueous extracts and their laxative pharmacological activity including the incubation period of defecation, the number of defecations, the wet weight of defecation, and the dry weight of defecation are shown in Figs. 9, 10, 11 and 12, respectively. Figure 9 shows that the presence of seven common peaks (P1, P2, P3, P5, P6, P8, and P9) in the HPLC fingerprint of *S. grosvenorii* aqueous extracts was highly correlated with the incubation period of defecation in mice (correlation grade > 0.90). The rank order for the contribution of each component to shortening the incubation period of defecation in mice was determined as P5 > P6, P9 > P2 > P1, P3, P8 > P4 > P7. Figure 10 shows that the presence of eight common peaks (P1, P2, P3, P4, P5, P6, P8, and P9) in the HPLC fingerprint of *S. grosvenorii* aqueous extracts was highly correlated with the number of defecations in mice (correlation grade > 0.80). The rank order for the contribution of each component to increasing the number of defecations in mice was determined as P5 > P2 > P6, P9 > P1, P3, P8 > P4 > P7. Figure 11 shows that the presence of seven common peaks (P1, P2, P3, P5, P6, P8, and P9) in the HPLC fingerprint of *S. grosvenorii* aqueous extracts was highly correlated with the effect of the wet weight of defecation in mice (correlation grade > 0.80). The rank order for the contribution of each component to increasing the wet weight of defecation in mice was determined as P5 > P2 > P6, P9 > P3 > P1, P8 > P4 > P7. Figure 12 shows that the presence of seven common peaks (P1, P2, P3, P5, P6, P8, and P9) in the HPLC fingerprint of *S. grosvenorii* aqueous extracts was highly correlated with the dry weight of defecation in mice (correlation grade > 0.80). The rank order for the contribution of each component to increasing the dry weight of defecation in mice was determined as P5 > P2 > P9 > P6 > P1, P3, P8 > P4 > P7. Of all the common peaks, P5 showed the highest correlation with the laxative pharmacological activity of the aqueous extracts. The only component whose identity was confirmed, P7 (mogroside V), which is part of the quality control index of *S. grosvenorii* in the Chinese Pharmacopoeia (2015 version), showed the worst correlation with the laxative pharmacological activity.

## Discussion

Functional constipation is one of the most common chronic functional gastrointestinal diseases in clinical treatment. It is believed that the pathogenesis of constipation is complicated and various, which mainly relates to colonic dysfunction or intestinal absorption of excessive moisture [26]. Therefore, the treatment strategies for functional constipation should focus on the promotion of intestinal motility as well as the prevention of excessive absorption of water. The pharmacological effects of phytomedicine on promoting defecation mainly includes: (1) shortening the incubation period of defecation, increasing the number or weight of defecation, softening the stool and improving the fecal water content, (2) enhancing the contraction frequency or amplitude of the ileum, and promoting the propulsion of the small and large intestines, and (3) increasing the water content of the enteric cavity or myenteron. In the current study, *S. grosvenorii* from some places of origin were observed to significantly shorten the incubation period of defecation, and significantly increase the number of defecations, and the wet and dry weights of defecation in constipated mice. These encouraging results suggested the definite curative effects of *S. grosvenorii* in treating constipation, which were equivalent or better than that of mosapride. We concluded that a potential explanation for the laxative effect of the aqueous extracts of *S. grosvenorii* was related to their ability to increase the MTL level in the serum of mice, and thus promote gastric emptying and intestinal propulsion. Further, it increased the water content of the small intestine, which supported its effect on bowel relaxation. Nevertheless, the mechanism by which *S. grosvenorii* increased the content of serum MTL and the water content of the small intestine in mice requires more detailed investigations of the gene and protein expression levels.

Fingerprint similarity evaluation is a method used to estimate the similarity of chemical components of phytomedicine. Theoretically, herbals with high similarities in their chemical fingerprints should have similar chemical components and thus, similar efficacies. We found that the fingerprints of different batches of *S. grosvenorii* were highly similar although the main characteristic peaks were not completely consistent. As shown in Fig. 2, nine common fingerprint peaks were identified from different batches. In the fingerprint, each characteristic peak represents a specific chemical component. The laxative effect of *S. grosvenorii* is the result of the synergistic action of its various chemical components. Hence, there must be some relationship between the fingerprint characteristics and the efficacy. However, the lack of a suitable method useful for identifying the components of *S. grosvenorii* that are efficacious is a critical gap in the quality control of *S. grosvenorii*. The use of simple bivariate correlation analysis and regression analysis are not applicable to explain the complex relationship between chemical composition and pharmacodynamics of *S. grosvenorii*. The gray correlation analysis provides an effective way to address this issue.

Gray relation analysis is a type of order relation analysis that has been successfully applied to decision-making, prediction, comprehensive evaluation, and accuracy testing of gray modeling. A large number of studies have confirmed that in research fields with less information and more interfering factors, gray relational analysis is one of the most promising mathematical approaches for order relation analysis. Currently, there are few studies on the application of gray correlation analysis in the quality evaluation of phytomedicine. In fact, this method is valuable because it uses known information to interpret unknown information. Therefore, it is suited for use in the quality evaluation and control of phytomedicine containing complex chemical components. As an exploratory analysis tool, gray correlation analysis provides a novel approach and shows great potential for standardization of *S. grosvenorii* for quality control purposes.

In the present work, we determined the contribution of the chemical components represented by the nine characteristic peaks of the fingerprint to the laxative effect of *S. grosvenorii* using gray correlation analysis. Since conventional research methods typically only allow the pharmacological effect of a single component or effective fractions of *S. grosvenorii* to be determined, the innovation of this work is in the establishment of a method useful to analyze the contribution of “effective component groups” to the laxative effect of *S. grosvenorii*. Considering that the effects of *S. grosvenorii* is not a simple linear superposition of its different components, in the future, we will use advanced analysis technology, powerful computer processing, and chemical information technology to “decode” the fingerprints, and further clarify the relationship between the “profile” and the “effect”. Additionally, except for Peak 7, we have not yet determined the specific chemical composition of the other eight common peaks. Especially, the structure and the phytochemical characteristic of Peak 5 are necessary to be elucidated, since it is the most relative one to the laxative effect. Through consulting related literatures [27, 28], we found that the main bioactive components of *S. grosvenorii* are a group of cucurbitane-type triterpene glycosides, normally named as mogrosides, mainly including mogroside III, mogroside IV, mogroside IVa, isomogroside V, mogroside VI, siamenoside I, mogroside V and 11-oxomogroside V. These mogrosides have mogrol triterpenoid aglycone in common and differ in the sugars attached. Therefore, we speculate that the other eight peaks may belong to a certain kind of the mentioned mogrosides. Future research will focus on the identification of these peaks.

## Conclusion

In the present study, nine common peaks were identified from the fingerprints of different batches of *S. grosvenorii* aqueous extracts by HPLC, making it a promising quality control method for *S. grosvenorii*. A pharmacological study of the laxative effect showed that *S. grosvenorii* aqueous extracts from 25 batches exhibited different therapeutic effects on mice with constipation because differences in their ecological environment, growth phase, breeding type and/or size fast influence the phytochemical profile resulting in different activities. Samples S4, S11, S14–S17, S20, and S23 obviously shortened the incubation period of defecation (*P* < 0.01), and significantly increased the number of defecations, the wet weight of defecation, and the dry weight of defecation in constipated mice (P < 0.01). The mechanism may be related to the improvement of the MTL serum level and the water absorption of the small intestine in normal mice, thus promoting gastric emptying and small intestinal peristalsis. With the help of gray correlation analysis, the profile-effect relationship between the nine common peaks of *S. grosvenorii* aqueous extracts and their laxative pharmacological activity was confirmed and the rank order for the contribution of each component was determined. This study is of great significance to the establishment of quality control standards as well as the determination of the effective components of *S. grosvenorii*.

## Supplementary Information

**Additional file 1.**

## Data Availability

The datasets from the present study are available from the corresponding author on reasonable request.

## Abbreviations

MTL: Motilin
HRP: Horseradish peroxidase
TMB: Tetramethylbenzidine
OD: Optical density
