# Supplementary material for: Exploration of the profile-effect relationship of Siraitia grosvenorii aqueous extracts related to their laxative effect on the basis of gray correlation analysis
Source: BMC Complement Med Ther. 2021 Sep 20;21:235. doi: 10.1186/s12906-021-03388-x (PMC8451111; doi:10.1186/s12906-021-03388-x)
Supplement: Supplementary file 1 — Additional file 1. [file 12906_2021_3388_MOESM1_ESM.pdf]

Table 1 Detail information of *S. Grosvenorii* samples

Samples	Regions	Growth phase	Breeding type	Fresh\ Dried	Size	GPS data (Lng)	GPS data (Lat)
S1	Yongfu County, Longjiang Township, Guangxi Province	40-45 days	#	-	unknown	109.838803	25.250554
S2	Yongfu County, Longjiang Township, Guangxi Province	60-70 days（colleted in Octomber）	#	-	unknown	109.838803	25.250554
S3	Yongfu County, Longjiang Township, Guangxi Province	40-45 days（colleted in Octomber）	*	-	unknown	109.838803	25.250554
S4	Yongfu County, Longjiang Township, Guangxi Province	60-70 days（colleted in Octomber）	*	-	unknown	109.838803	25.250554
S5	Yongfu County, Longjiang Township, Guangxi Province	89-90 days（colleted in Octomber）	#	-	medium	109.838803	25.250554
S6	Yongfu County, Longjiang Township, Guangxi Province	ripe（colleted in Octomber）	*	-	medium	109.838803	25.250554
S7	Yongfu County, Longjiang Township, Guangxi Province	ripe（colleted in Octomber）	*	-	big	109.838803	25.250554
S8	Yongfu County, Longjiang Township, Guangxi Province	60-70 days（colleted in November）	#	-	unknown	109.838803	25.250554
S9	Yongfu County, Longjiang Township, Guangxi Province	ripe（colleted in Octomber）	#	-	medium	109.838803	25.250554
S10	Yongfu County, Longjiang Township, Guangxi Province	ripe（colleted in November）	#	+	medium	109.838803	25.250554
S11	Yongfu County, Longjiang Township, Guangxi Province	ripe（colleted in November）	*	-	medium	109.838803	25.250554
S12	Yongfu County, Longjiang Township, Guangxi Province	ripe（colleted in November）	*	-	big	109.838803	25.250554
S13	Yongfu County, Longjiang Township, Guangxi Province	ripe（colleted in November）	*	-	fruitlet	109.838803	25.250554

S14	Yongfu County, Longjiang Township, Guangxi Province	ripe（colleted in November）	*	-	medium	109.838803	25.250554
S15	Yongfu County, Longjiang Township, Guangxi Province	ripe（colleted in November）	*	-	medium	109.838803	25.250554
S16	Yongfu County, Longjiang Township, Guangxi Province	ripe	*	-	medium	109.838803	25.250554
S17	Yongfu County, Longjiang Township, Guangxi Province	ripe（colleted in November）	#	-	medium	109.838803	25.250554
S18	Hengyang, Hunan Province	ripe（colleted in Nov）	unknown	fresh fruits	medium type	112.574425	26.898286
S19	Hengyang, Hunan Province	ripe（colleted in Nov）	unknown	dry fruits	medium type	112.574425	26.898286
S20	Nanning Academy of Agricultural Sciences, Guangxi Province	ripe（colleted in Octomber）	unknown	+	medium	108.255574	22.847334
S21	Nanning Academy of Agricultural Sciences, Guangxi Province	ripe	unknown	-	medium	108.255574	22.847334
S22	Yongfu County, Baoli Township, Guangxi Province	ripe（colleted in December）	*	+	fruitlet	110.078866,	24.853251
S23	Yongfu County, Baoli Township, Guangxi Province	ripe（colleted in December）	*	+	medium	110.078866	24.853251
S24	Yongfu County, Baishou Town, Guangxi Province	ripe（colleted in November）	#	-	medium	109.77796	25.162092
S25	Yongfu County, Baishou Town, Guangxi Province	ripe（colleted in November）	*	-	medium	109.77796	25.162092

Note: “#” means tissue culture, “*” means cottage, “+” means Fresh, “-” means Dried.
